# Yes, Bridges Do Connect!—A Rare Case of Coronary Sinus Communicating Into the Left Atrium Through a Bridging Vein

**DOI:** 10.1002/ccr3.70961

**Published:** 2025-09-24

**Authors:** Manisha Aryal, Nirmal Prasad Neupane, Kritisha Rajlawot, Ishwor Paudel, Sandeep Koirala, Suraj Keshari, Archana Pandey

**Affiliations:** ^1^ Shahid Gangalal National Heart Center Kathmandu Nepal; ^2^ Bir Hospital Kathmandu Nepal; ^3^ Paropakar Maternity and Women's Hospital Kathmandu Nepal; ^4^ Kathmandu University School of Medical Sciences Dhulikhel Hospital Dhulikhel Nepal

**Keywords:** anomalous venous drainage, bridging vein, case report, computed tomography‐coronary angiography, coronary sinus

## Abstract

Coronary sinus (CS) anomalies are usually asymptomatic and are incidentally discovered on cardiac imaging or autopsy. Failure to recognize coronary sinus anomalies may give rise to misinterpretations of cardiac catheterization data, and the associated altered hemodynamics may lead to troublesome effects during surgical procedures.

## 1 Introduction

The coronary sinus is the large venous structure located in the posterior aspect of the heart. Understanding the normal anatomy and anomalies of the coronary sinus (CS) and cardiac venous drainage is essential in avoiding complications related to cardiac surgeries and electrophysiological treatments [1, 2, 3]. A gross connection between the coronary sinus and the left atrium is a rare occurrence, which, in some cases, may be functionally significant [4]. Anomalies of the coronary sinus and the cardiac venous system can occur in isolation or in association with other congenital heart defects [5]. Failure to recognize coronary sinus anomalies may give rise to severe misinterpretations of cardiac catheterization data, and the associated altered hemodynamics may lead to troublesome effects during surgical procedures [6, 7]. Cardiac CT and MRI provide excellent anatomical details of the coronary sinus, avoiding invasive diagnostic methods. Therapeutic intervention can be approached, without any doubt, with the use of these non‐invasive imaging modalities [8].

In this case report, we present a female in her 50s with an abnormal communication of the coronary sinus with the left atrium through a bridging vein. We also review the current literature on this condition and discuss her CT‐CAG (computed tomography‐coronary angiography) findings in detail. This case has been documented for its rarity and educational value.

## 2 Case History

A female patient aged 57 years presented to the outpatient department of our tertiary cardiac center with complaints of acute onset of sudden awareness of her heartbeat since 4–6 h. She described the palpitations as irregular, fast, and persistent and reported that they were accompanied by sweating and dizziness. She also gives a history of chest pain and chest heaviness for the past 4 h, which was also of acute onset. The chest pain was dull in nature, localized to the central chest region, and non‐radiating. Importantly, the chest pain did not worsen with exertion. She had a history of hypertension and was on anti‐hypertensive medication for 10 years. Otherwise, the patient's medical and surgical history is unremarkable. The patient did not have any history of trauma or intervention in the past. No similar conditions or cardiovascular illnesses have been reported in the family history. Additionally, the patient denies any history of smoking, alcohol consumption, or recreational drug use.

On cardiovascular examination, normal heart sounds (S1 and S2) were heard with no murmurs. No signs of edema or jugular venous distension were found. Systemic examinations, including the gastrointestinal, respiratory, and neurological systems, detected no abnormalities.

## 3 Methods

Laboratory investigations, electrocardiogram, and chest radiographs were unremarkable. An electrocardiogram showed normal sinus rhythm without any ST or T wave changes. A transthoracic echocardiogram showed normal function of the left ventricle, a mildly dilated right atrium, no significant valvular pathology, pulmonary systolic pressure (PASP) of 27 mmHg, and Grade I diastolic dysfunction. A computed tomography coronary angiography (CT‐CAG) was further advised for the assessment of coronary vessels.

**FIGURE 1:**
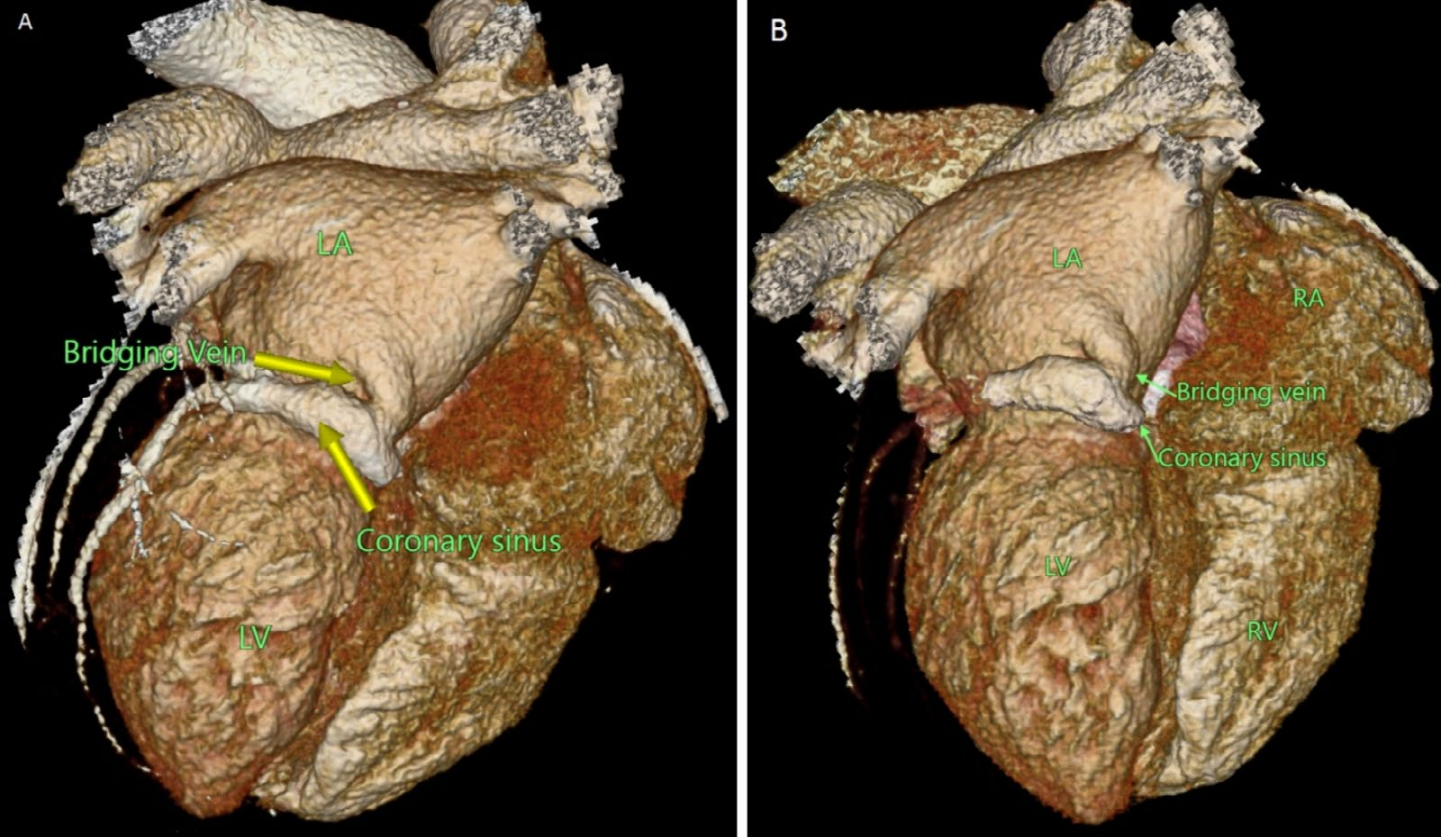
(A, B) 3‐D reconstruction showing the coronary sinus communicating with the left atrium through the bridging vein (LA, left atrium; LV, left ventricle; RA, right atrium; RV, right ventricle).

**FIGURE 2:**
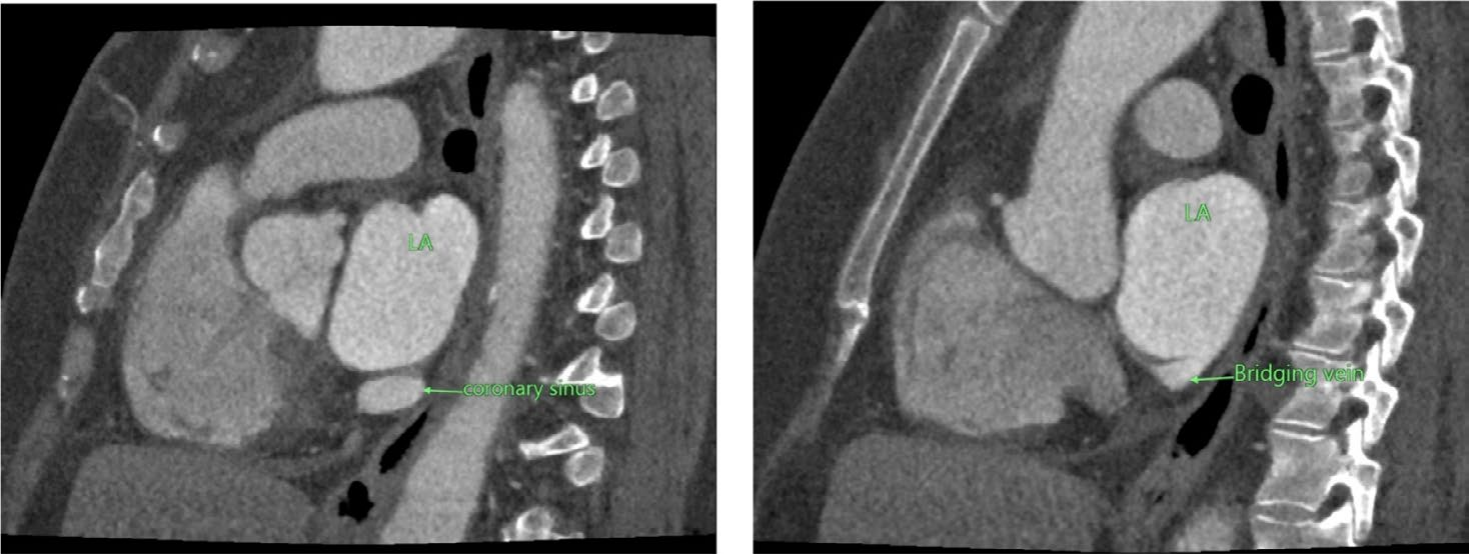
Computed tomography coronary angiogram sagittal views showing the indirect communication of the coronary sinus and left atrium through a bridging vein.

**FIGURE 3:**
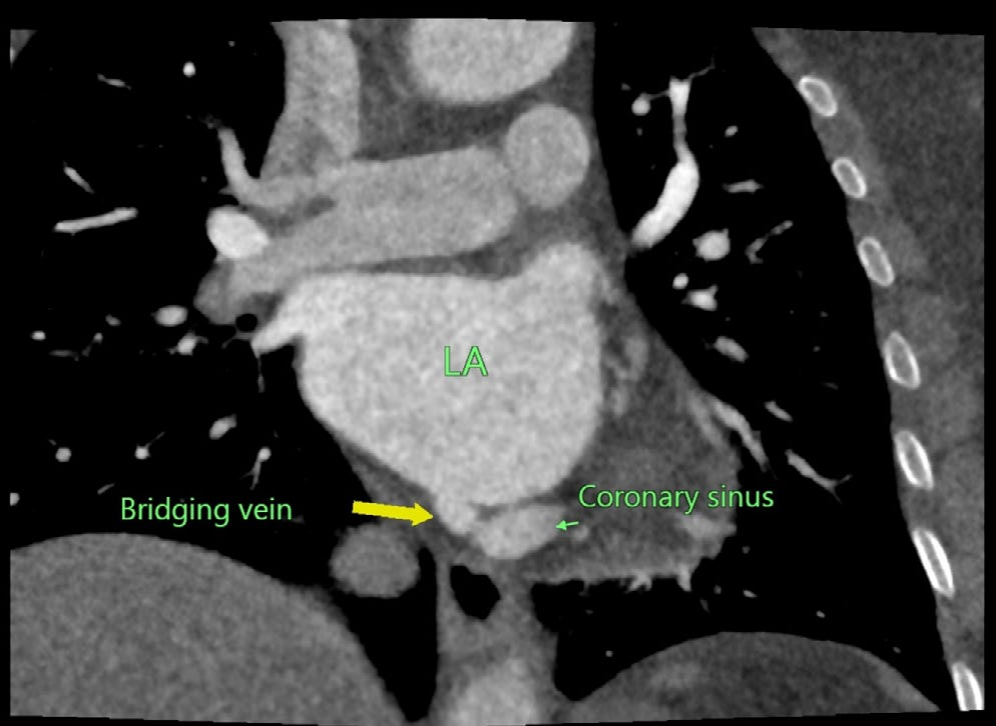
Coronal CT angiogram showing the coronary sinus draining into the left atrium through a bridging vein.

CT‐CAG revealed the total coronary artery calcium score of 46. There was a normal origin of the left main coronary artery from the left sinus of Valsalva, further branching into the left anterior descending artery (LAD) and left circumflex artery (LCX). The normal origin of the right coronary artery from the right sinus of Valsalva with normal branching and terminations was noted. No significant stenosis of the coronary arteries was noted. Normal drainage of the coronary sinus into the right atrium was not appreciated. The coronary sinus was noted to communicate with the left atrium through a bridging vein in Figures 1, 2, 3.

### 3.1 Outcome and Follow‐Up

Since the patient is asymptomatic like most cases of coronary sinus anomalies, she does not require surgical management. However, we plan to monitor the patient with follow‐ups every 6 months. Each follow‐up will include a clinical examination and electrocardiogram, with echocardiography at 1 year intervals.

## 4 Discussion

The coronary sinus is a venous structure of the heart that drains blood from the great cardiac vein, the middle cardiac vein, and other tributary veins. The ostium of the coronary sinus lies in the right atrium of the heart [9, 10]. During ventricular systole, the coronary sinus receives blood from ventricular veins, and during atrial systole, it empties into the right atrium [11]. Anomalies of the coronary sinus are classified into four anatomic groups: (1) on the basis of coronary sinus enlargement, (2) absence of the coronary sinus, (3) atresia of the right atrial coronary sinus ostium, and (4) coronary sinus hypoplasia [5]. Enlargement of the CS may occur in a variety of pathologic situations, including congestive heart failure [12]. In our case, it was due to the abnormal connection between the coronary sinus and the left atrium.

A coronary arterio‐venous fistula is associated with a high‐pressure left‐to‐right shunt, and an anomalous communication of the coronary sinus with the LA or pulmonary venous system is associated with a low‐pressure left‐to‐right shunt. The coronary sinus may communicate with the LA either indirectly through a bridging vein between the two structures or directly through a wall defect between the coronary sinus and the left atrial cavity [5]. We observed indirect communication in our case, as shown by CT. The etiology of this defect may be congenital, cardiac trauma, or iatrogenic [12]. The patient in our case did not have any history of trauma or intervention in the past.

Usually, coronary sinus anomalies are asymptomatic and are incidentally discovered on cardiac imaging or autopsy. However, the degree of symptoms in coronary sinus anomalies can be variable and depends mainly on the volume and direction of the shunt. Some may present with symptoms of right heart failure or persistent atrial fibrillation despite pulmonary vein isolation [13]. However, in our case, the patient's acute onset palpitations, dizziness, and sweating can be partially related to the hemodynamic effects of an anomalous communication between the coronary sinus and the left atrium. This abnormal communication can result in a right‐to‐left shunt, especially during periods of elevated right atrial pressure (e.g., physical exertion or transient volume overload) [14].

The palpitations may be linked to atrial irritation or volume changes due to the anomalous flow, although no arrhythmia was documented at the time of evaluation.

On the imaging side, coronary CT with delayed phase, MRI, and invasive angiography are the best tools for diagnosing coronary sinus anomalies [4, 13, 15]. In symptomatic cases of coronary sinus anomalies, coronary sinus ablation may be considered. However, most cases are asymptomatic and may not require intervention [14], similar to our patient's case.

In conclusion, coronary sinus anatomy and the wide spectrum of its variants and anomalies can be studied in detail with the help of coronary CT angiography. While most patients having a coronary sinus to left atrium shunt via a bridging vein are asymptomatic, like our case, coronary sinus ablation may be necessary in symptomatic patients to prevent atrial fibrillation. So, clear anatomical details of the heart and its vessels should be described in the CT report, as this information is essential for determining the appropriate treatment for patients.

## Author Contributions

**Manisha Aryal:** conceptualization, data curation, supervision, writing – original draft, writing – review and editing. **Nirmal Prasad Neupane:** data curation, writing – original draft, writing – review and editing. **Kritisha Rajlawot:** data curation, writing – original draft, writing – review and editing. **Ishwor Paudel:** writing – original draft, writing – review and editing. **Sandeep Koirala:** writing – original draft, writing – review and editing. **Suraj Keshari:** writing – original draft, writing – review and editing. **Archana Pandey:** writing – original draft, writing – review and editing.

## Ethics Statement

The authors have nothing to report.

## Consent

Written informed consent was obtained from the patient for publication of this case report and accompanying images. A copy of the written consent is available for review by the Editor‐in‐Chief of this journal on request.

## Conflicts of Interest

The authors declare no conflicts of interest.

## Data Availability

Data sharing not applicable to this article as no datasets were generated or analyzed during the current study.
